# Chemical Mechanism of UDP-Galactopyranose Mutase from *Trypanosoma cruzi*: A Potential Drug Target against Chagas' Disease

**DOI:** 10.1371/journal.pone.0032918

**Published:** 2012-03-20

**Authors:** Michelle Oppenheimer, Ana Lisa Valenciano, Karina Kizjakina, Jun Qi, Pablo Sobrado

**Affiliations:** 1 Department of Biochemistry, Virginia Tech, Blacksburg, Virginia, United States of America; 2 Enzyme Research and Drug Discovery Laboratory, Virginia Tech, Blacksburg, Virginia, United States of America; 3 Instituto Tecnológico de Costa Rica, Cartago, Costa Rica; 4 Fralin Life Science Institute, Virginia Tech, Blacksburg, Virginia, United States of America; National Institutes of Health, United States of America

## Abstract

UDP-galactopyranose mutase (UGM) is a flavoenzyme that catalyzes the conversion of UDP-galactopyranose to UDP-galactofuranose, the precursor of galactofuranose (Gal*f*). Gal*f* is found in several pathogenic organisms, including the parasite *Trypanosoma cruzi*, the causative agent of Chagas' disease. Gal*f*) is important for virulence and is not present in humans, making its biosynthetic pathway an attractive target for the development of new drugs against *T. cruzi*. Although UGMs catalyze a non-redox reaction, the flavin must be in the reduced state for activity and the exact role of the flavin in this reaction is controversial. The kinetic and chemical mechanism of TcUGM was probed using steady state kinetics, trapping of reaction intermediates, rapid reaction kinetics, and fluorescence anisotropy. It was shown for the first time that NADPH is an effective redox partner of TcUGM. The substrate, UDP-galactopyranose, protects the enzyme from reacting with molecular oxygen allowing TcUGM to turnover ∼1000 times for every NADPH oxidized. Spectral changes consistent with a flavin iminium ion, without the formation of a flavin semiquinone, were observed under rapid reaction conditions. These data support the proposal of the flavin acting as a nucleophile. In support of this role, a flavin-galactose adduct was isolated and characterized. A detailed kinetic and chemical mechanism for the unique non-redox reaction of UGM is presented.

## Introduction

The protozoan parasite *Trypanosoma cruzi* is the etiological agent of American trypanosomiasis or Chagas' disease. The World Health Organization estimates that 8–11 million people are infected with this disease in South and Central America [1]. Transmission occurs when a triatomine insect feeds on a human subject and defecates at the bite site [2]. Through rubbing, infected feces enter the bite wound, mouth, eyes, or open cuts. Transmission by blood transfusion, organ transplant, oral contamination, and congenital routes has also been reported [3]–[7]. *T. cruzi* has a dynamic life cycle, involving several morphological changes as the parasites travel from the insect vector to humans [8]. This is accompanied by several changes in cell surface sugar composition, which plays an important role in infection and resistance to the host immune system [9], [10]. Targeting the enzymes involved in biosynthesis of cell surface glycans may lead to the identification of new inhibitors that function as novel antiparasitic drugs for the treatment of Chagas' disease [11]. One unique sugar found on the cell surface of *T. cruzi* is galactofuranose (Gal*f*) [11].

**Figure 1:**
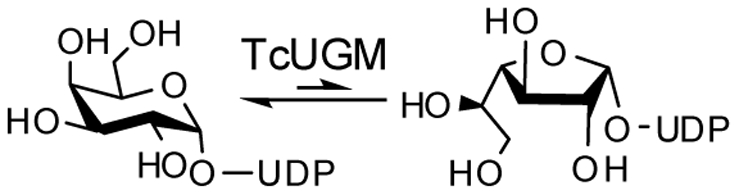
Reaction catalyzed by TcUGM.

**Figure 2:**
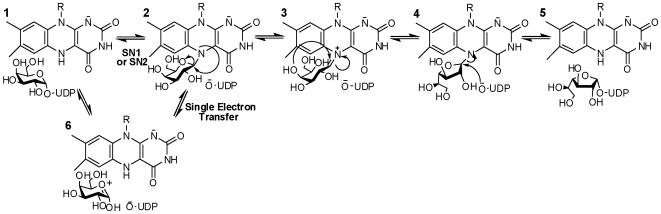
The two proposed chemical mechanisms for UGMs. In one mechanism the reduced FAD (**1**) is depicted to act as a nucleophile forming a flavin-galactose adduct (either via S_N_1 or S_N_2) (**2**) and a subsequent iminium ion (**3**). These steps are followed by ring contraction forming the galactofuranose (**4**). An alternative mechanism predicts an electron transfer step, in which one electron is transferred from FADH^−^ to an oxocarbenium ion intermediate (**6**) creating a flavin and a sugar radical, which react and form the galactose-FAD adduct (**2**), followed by ring contraction.

Gal*f*, the five-membered ring form of galactose, is a component of the cell wall, glycolipids, and glycoproteins on the cell surface of many human pathogens including bacteria, fungi, and parasites [11]–[14]. In *T. cruzi*, Gal*f* is found in glycoprotein oligosaccharides and glycoinositolphospholipids, which are involved in parasite pathogenesis [11], [15], [16]. Additionally, Gal*f* is not present in humans. Thus, the biosynthetic pathway of Gal*f* is an attractive drug target for *T. cruzi* and other eukaryotic pathogens including *A. fumigatus* and *L. major* [11], [17]. UDP-galactopyranose mutase (UGM) catalyzes the conversion of UDP-galactopyranose (UDP-Gal*p*) to UDP-galactofuranose (UDP-Gal*f*), the precursor of Gal*f* found on the cell surface (Figure 1) [18]. UGM is a unique flavoprotein, as it requires the flavin to be reduced in order to catalyze a non-redox reaction (Figure 2) [19], [20]. The role of the flavin cofactor in catalysis is controversial. Experimental and structural data supports the role of the flavin acting as a nucleophile [21], [22]. Similarly, studies with flavin analogs and potentiometry experiments suggest that a single electron transfer step is necessary for catalysis [23], [24]. Here, we present a complete characterization of the recombinant form of UGM from *T. cruzi* (TcUGM). We utilize steady state kinetics, fluorescence anisotropy, rapid reaction kinetics, and the trapping of reaction intermediates to provide a clear view of the kinetic and chemical mechanisms employed by this unique enzyme. We also identify NAD(P)H as an effective electron donor to TcUGM, a function that is unique to eukaryotic UGMs.

## Materials and Methods

### Materials

UDP, UDP-galactopyranose, and BL21-T1^R^ chemical competent cells were purchased from Sigma (St. Louis, MO). Accuprime polymerase and TOP-10 chemical competent cells were obtained from Invitrogen (Carlsbad, CA). The restriction endonucleases *Sgf*I and *Pme*I were obtained from Promega (Madison, WI). Plasmid miniprep and PCR purification kits were from Qiagen (Valencia, CA). All other buffers and chemicals for biochemical studies were purchased from Fisher Scientific (Pittsburgh, PA). Expression plasmids, pVP55A and pVP56K, were obtained from the Center for Eukaryotic Structural Genomics, University of Wisconsin, Madison [25]. All reagents and solvents used in the synthesis of UDP-Gal*f*, if not specified otherwise, were obtained from Sigma-Aldrich (St. Louis, MO) or Fisher Scientific (Pittsburgh, PA) and were used without further purification. All the synthetic reactions were monitored to completion using thin layer chromatography (TLC) with *p*-anisaldehyde staining for visualizing carbohydrates along with UV detection when possible. ^1^H and ^13^C NMR were recorded on a 400MR Varian-400 MHz spectrometer. CHCl_3_ (7.27 ppm) or HOD (4.79 ppm) were used as an internal reference.

### Cloning

*T. cruzi* UGM (TcUGM) was amplified by PCR from genomic DNA using 5′-GGTTGCGATCGCCATGGCAGAATTATTGACACC -3′(*Sgf*I site is underlined) as the forward primer and 5′- CCAAGTTTAAACCATATCCTTCTGCAGTAGTC -3′ (*Pme*I site is underlined) as the reverse primer. The TcUGM gene was inserted into the pVP55A and pVP56K vectors as previously described [17].

### Protein expression and purification

Protein expression and purification of TcUGM was performed following methods previously described for *A. fumigatus* UGM with an additional final step of size exclusion chromatography in 25 mM HEPES, 125 mM NaCl, pH 7.5 (S-75, GE Healthcare, Piscataway, NJ) [17]. Purified TcUGM was concentrated, flash frozen in liquid N_2_, and stored at −80°C.

### UV–visible absorbance spectrophotometry

The spectrum of recombinant TcUGM was recorded using an Agilent 8453 UV–visible spectrophotometer. The extinction coefficient was determined by dividing the absorbance value at 450 nm of the bound flavin in TcUGM by the absorbance value at 450 nm of free flavin (obtained by heat denaturation and centrifugation of the recombinant enzyme) and multiplying this value by the known extinction coefficient for FAD (ε_FAD_ = 11.3 mM^−1^cm^−1^) [26].

### Solution molecular weight determination

The molecular weight of TcUGM was determined using size exclusion chromatography as previously described [17], [27].

### NAD(P)H oxidation assays

Oxidation of NAD(P)H was monitored at 340 nm for 5 min. Reactions were performed at room temperature with air saturated 50 mM sodium phosphate buffer, pH 7.0, with various concentrations of NAD(P)H, in the presence or absence of 0.5 mM UDP-Gal*p*. The reaction was initiated by addition of 1 µM enzyme.

### Flavin reduction by NAD(P)H

Flavin reduction by NAD(P)H was performed anaerobically in a glove box (Coy, Grass Lake, MI) using an Applied Photophysics Stopped Flow SX20 (Leatherhead, UK). Reactions were performed at 15°C. Buffer (50 mM sodium phosphate pH 7.0) was made anaerobic by eight cycles of vacuum and argon flushing, each for 45 minutes. After this procedure, protocatechuate (400 µM) and protocatechuate dioxygenase (10 µg/mL) were added to the buffer to function as an oxygen scavenging system. All the solutions were prepared with the anaerobic buffer in the glove box. NAD(P)H solutions were prepared by dissolving the appropriate amounts in anaerobic buffer and concentrations were verified spectroscopically. The enzyme solution was made anaerobic by degassing with six 15 min cycles of vacuum and flushing with anaerobic argon. To ensure complete anaerobiosis, the solution was passed through a 2 mL desalting column previously equilibrated with anaerobic buffer. Enzyme with a final concentration of ∼7.5 µM (after mixing) was mixed with various concentrations of NAD(P)H and the reaction was monitored with a photodiode array spectrophotometer until complete reduction was achieved. Identical experiments were performed in the presence of 0.5 mM UDP-Gal*p*. Change in absorbance at 452 nm was fitted to a single exponential equation and the resulting k*_obs_* values were plotted as a function of NAD(P)H concentration. These data was fit with equation 1 to obtain the rate constant for reduction (*k_red_*) and the *K_d_* value.
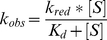
(1)


### Synthesis of UDP-Gal*f*

Synthesis of α-d-Galactofuranosyl bis(triethylammonium) phosphate (Gal*f*-1-phosphate) was performed over a series of three steps with methods adapted from previously published protocols [28]–[34]. Details of the synthesis and NMR data are outlined in the supplementary material (File S1). UDP-Gal*f* was synthesized following published method reported by Poulin and coworkers [35].

### Activity assay

The activity of recombinant TcUGM was tested with UDP-Gal*f* as the substrate following procedures previously described [17]. Concentration of TcUGM was determined based on bound flavin. TcUGM (100 nM) was reduced with either 20 mM dithionite, 500 µM NADPH, or 2.5 mM NADH for each activity assay.

### Viscosity Effects

Viscosity effects were determined using the activity assay as described above. Reactions were done in the presence of 0, 5, 10, 20, and 30% glycerol, 800 µM UDP-Gal*f*, 20 mM dithionite, and 200 nM TcUGM. The relative viscosity (η_rel_) of the reaction was determined using a reference table for different percentages of glycerol in solution [36].

### Fluorescence anisotropy

Synthesis of UDP-rhodamine was performed as previously described [37]. The optimal concentration of TcUGM (30 µM) and UDP-rhodamine (30 nM) for the binding assays was determined following procedures established in our laboratory for a related UGM enzyme from *Aspergillus fumigatus* [37]. Binding of UDP-Gal*p* to TcUGM was monitored in the presence and absence of 10 mM sodium dithionite by measuring the changes in anisotropy [37]. The K*_d_* values were obtained using the equation 2, where m_1_ and m_2_ are the minimum and maximum anisotropy, respectively; m_3_ is the slope, and m_4_ is the K*_d_*.
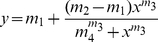
(2)


### Trapping of the flavin-galactose adduct

The procedure was adapted from Gruber *et al.* and optimized for trapping and isolation of the adduct in TcUGM [22]. Reactions were prepared using 1 mM TcUGM, 200 mM UDP-Gal*p*, 1 M sodium cyanoborohyride, and 100 mM sodium dithionite in a final volume of 15 µL with 100 mM sodium phosphate buffer at pH 7.0. Reactions were incubated for 45 min at room temperature then terminated with addition of 6 M HCl and neutralized with 1 M sodium phosphate buffer at pH 7.0. A final concentration of 200 mM NaCl was added. The sample was then centrifuged to precipitate denatured protein from the yellow solution containing the free flavin. The adduct was isolated using HPLC (Shimadzu) by injecting the yellow solution onto a reverse phase C18 column (Phenomenex Luna C18 5 microns, 250×4.6 mm) equilibrated with 5% B (A: Water, B: 100% acetonitrile). The column was washed for 5 min with 5% B, then a 15 min linear gradient to 15% B, followed by 20 min linear gradient to 75% B. The flavin species were eluted at ∼20–24 minutes and were characterized by high-resolution mass spectrometry (Virginia Tech Mass Spectrometry incubator).

### Reaction of reduced TcUGM with UDP-Gal*p*, UDP, and UDP-glucose

Rapid reaction kinetics were done using the methods described for the TcUGM reduction studies. However, reduced TcUGM was prepared by addition of 20 mM sodium dithionite. Excess dithionite was removed using a 2 mL desalting column. Reduced TcUGM (∼15 µM final concentration) was mixed with buffer alone for a final concentration of 0.15 mM UDP-Gal*f*, 0.25 mM UDP-Gal*p*, 0.25 mM UDP, and 0.25 mM UDP-glucose (UDP-Glc). Spectra were collected on a logarithmic time base from 1.3 ms to 2 s using a photodiode array. The rate of the intermediate formation was calculated by fitting the change in absorbance at 452 nm to a double exponential equation 3.
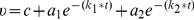
(3)


## Results

### Expression and Purification

Successful expression of soluble TcUGM was accomplished in the pVP56K vector [17]. The protein was expressed as an N-terminal fusion to maltose binding protein (MBP) with an 8×-His tag, which aids in purification. BL21-T1^R^ cells containing the pVP56K-TcUGM plasmid were grown in auto-induction medium for 8 h at 37°C followed by overnight incubation at 25°C [38]. Approximately 9 g of cell paste per liter of media was obtained and 1 mg of purified TcUGM was isolated for each g of cell paste. Recombinant TcUGM contained noncovalently bound flavin cofactor, which had a typical flavin spectrum with peaks at 376 nm and 448 nm with a shoulder at 468 nm (Figure S1). The extinction coefficient was determined to be 10.8 mM^−1^ cm^−1^ at 450 nm.

### Determination of the oligomeric state

Size exclusion chromatography was used to calculate a molecular weight of 67,800±10,000 Da for the recombinant TcUGM (Figure S2). This value is consistent with the molecular weight predicted for a single polypeptide (54,700 Da).

### Oxidase activity with NADPH and NADH

**Figure 3:**
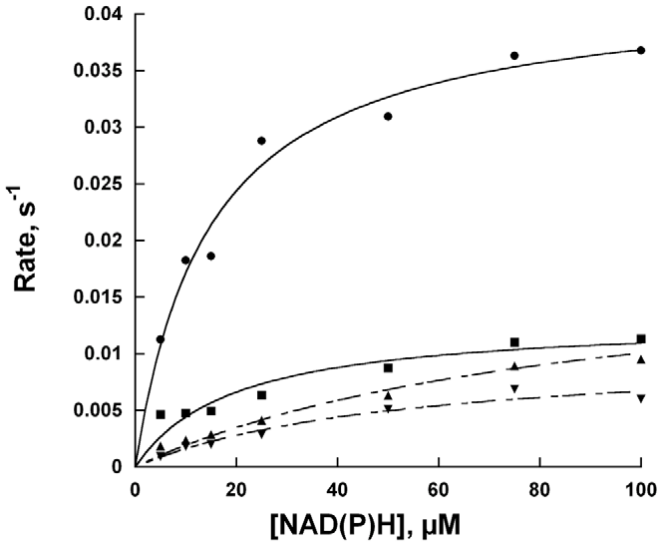
Oxidase activity of TcUGM with NAD(P)H. A) Scheme representing the oxidase activity of TcUGM measured in this assay. B) Activity of TcUGM measured in the presence and absence of saturating concentration of UDP-Gal*p* (0.5 mM) under air saturating conditions at room temperature (NADPH (•), NADH (▴), NADPH+UDP-Gal*p* (▪), and with NADH+UDP-Gal*p* (▾)).

The activity of all UGMs requires the flavin to be in the reduced state. Although the prokaryotic enzymes have been extensively studied, the mechanism of reduction is yet to be determined [20], [39]. Even though an NAD(P)H binding domain was not found in the primary sequence of TcUGM, we tested whether this enzyme was capable of reacting with reduced dinucleotides. The oxidation of either NADH or NADPH was monitored in the presence and absence of UDP-Gal*p* under aerobic conditions. Under this conditions, no mutase activity is measured, instead what is measured is the steady-state reduction of the flavin cofactor in TcUGM, which is slowly oxidized by molecular oxygen (Figure 3). In the absence of UDP-Gal*p*, a slow oxidase activity, k*_ox_*, was observed for both coenzymes, while a 6-fold lower K*_M_* value was measured for NADPH. In the presence of UDP-Gal*p*, a minor decrease in k*_ox_* was measured (Table 1). In contrast, when the same activity was measured with recombinant *M. tuberculosis* UGM (MtUGM), there was no measurable NAD(P)H oxidation (Table 1). *E. coli* UGM has also been shown to be unable to react with NAD(P)H [20], [39].

**Table 1:** Kinetic parameters of NAD(P)H oxidation reactions^a^.

	NADH		NADPH	
	k*_ox_*, s^−1^	K*_M_*, µM	k*_ox_*, s^−1^	K*_M_*, µM
TcUGM	0.015±0.002	90±20	0.030±0.002	15±2
TcUGM+UDP-Gal*p* b	0.010±0.002	50±20	0.0099±0.001	19±5
MtUGM	1.8×10^−6^	-	1.1×10^−6^	-
NAD(P)H alone	1.8×10^−7^	-	4.7×10^−7^	-

a All reactions were done at room temperature with air saturated buffer (50 mM phosphate buffer pH 7.0).

b In the presence of 0.5 mM UDP-Gal*p*.

### Flavin reduction monitored by rapid reaction kinetics

**Figure 4:**
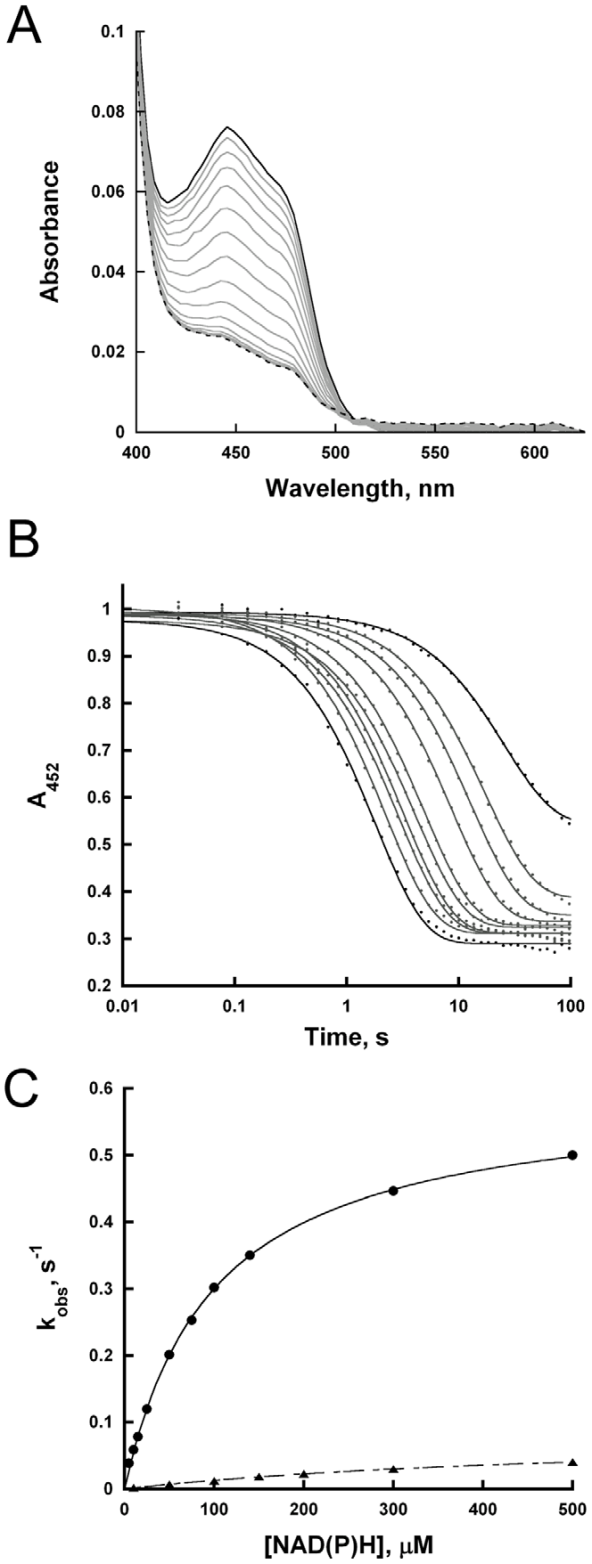
Anaerobic reduction of TcUGM with NAD(P)H. Reduction was monitored using the stopped flow spectrophotometer at 15°C. The data was collected from 2 ms to 100 s on a logarithmic timescale. A) Changes in the spectra of oxidized TcUGM after mixing with 0.5 mM NADPH over 23 s. B) Traces of the flavin reduction at various concentrations of NADPH. The data were fit to a single exponential decay equation. C) The k*_obs_* values were plotted as a function NADPH (•) and NADH (▴) concentrations and fitted using the equation 2.

The decrease in absorbance at 450 nm under anaerobic conditions was measured in a stopped flow spectrophotometer to determine the rate constant of flavin reduction of TcUGM by the reduced coenzyme (Figure 4). The observed rate constant of flavin reduction, k*_red_*, with NADPH was seven-fold faster than with NADH (Table 2, Figure 4). Furthermore, the K*_d_* value for NADPH was five-fold lower than for NADH. These results suggest that NADPH is the preferred coenzyme. Reduction of TcUGM with NADPH was also tested in the presence of saturating UDP-Gal*p*. Under this condition, the k*_red_* value did not change, demonstrating reduction is unaffected by the presence of UDP-Gal*p* (0.57±0.01 s^−1^ in the absence of UDP-Gal*p* versus 0.57±0.04 s^−1^ in the presence of UDP-Gal*p*).

**Table 2:** TcUGM Reduction by NAD(P)H^a^.

	^k^ *_red_*, s^−1^	^K^ *_d_*, µM	*k_red_*/K*_d_*, µM^−1^ s^−1^
**^NADPH^**	0.60±0.006	98±3	0.0061±0.0001
**^NADH^**	0.085±0.0006	550±10	0.00015±0.000002

a Reactions were measured under anaerobic conditions at 15°C in 50 mM phosphate buffer pH 7.0.

### Mutase activity

**Figure 5:**
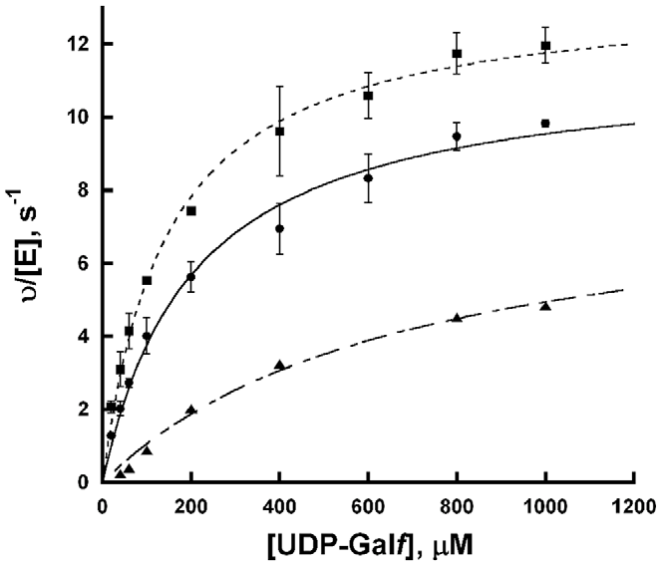
TcUGM activity with UDP-Gal*f*. TcUGM was reduced with either 20 mM dithionite (▪), 0.5 mM NADPH(•), or 2.5 mM NADH(▴). Reactions were performed with 200 nM TcUGM incubated with varying concentrations of substrate for 1 min at 37°C. The data were fit to the Michaelis-Menten equation. Summary of data is presented in Table 3.

The activity of TcUGM reduced with NADPH and NADH was measured and compared to the values obtained with dithionite reduced enzyme (Table 3, Figure 5). The reverse reaction was monitored to allow significant accumulation of product, as equilibrium favors UDP-Gal*p* over UDP-Gal*f* (93% to 7%). Saturation kinetics was observed as a function of UDP-Gal*f* concentration. Data were fit to the Michaelis-Menten equation and very similar k*_cat_* values were obtained independent of the source of reducing equivalents (Table 3). The K*_M_* values were also very similar when the enzyme was reduced with NADPH or dithionite. In contrast, a higher K*_M_* value was measured with NADH as the reductant, which we attributed to the slower rate of reduction by NADH (Table 3).

**Table 3:** Steady state kinetics of TcUGM^a^.

Reductant	k*_cat_*, s^−1^	K*_M_*, µM	k*_cat_*/K*_M_*, µM^−1^ s^−1^
Dithionite	13.4±0.3	140±10	0.093±0.006
NADPH	11.5±0.4	200±20	0.056±0.005
NADH	8.4±0.9	690±150	0.012±0.001

a Reactions were incubated at 37°C for 1 min in 25 mM Hepes, 125 mM NaCl pH 7.5, and terminated by heat denaturation at 95°C. Reactions were performed using saturating amounts of reductant (20 mM dithionite, 0.5 mM NADPH, and 2.5 mM NADH).

### Viscosity effects

**Figure 6:**
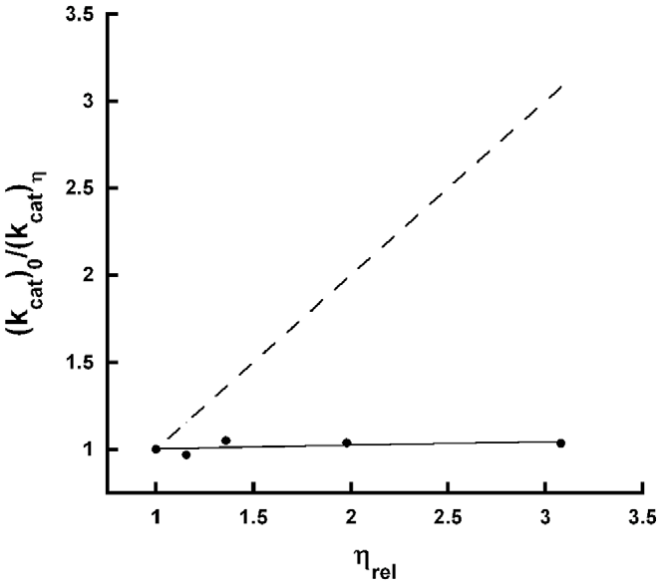
Viscosity effect on k_cat_. The effect of viscosity was determined by measuring the activity of TcUGM as a function of increasing concentrations of glycerol. The data was fit to a linear equation; the dashed line depicts the results of a diffusion controlled reaction. This line has a slope of 1.

To determine if product release is the rate-limiting step, the effect of viscosity on k*_cat_* was measured. This was done by determining the activity of TcUGM at different concentrations of glycerol. If product release was the rate-limiting step, a decrease in the k*_cat_* value would be observed as the viscosity of the medium increased. A plot of the ratio of the k*_cat_* values in water, (k*_cat_*)_0_, and at each concentration of viscogen, (k*_cat_*)_η_, should yield a straight line with a positive slope. A slope of 1 is expected if product release is the rate-liming step. With TcUGM, a slope of zero was observed, indicating that product release is not the rate determining step in the reaction (Figure 6).

### Determination of the binding affinity of UDP-Gal*p* to TcUGM

**Figure 7:**
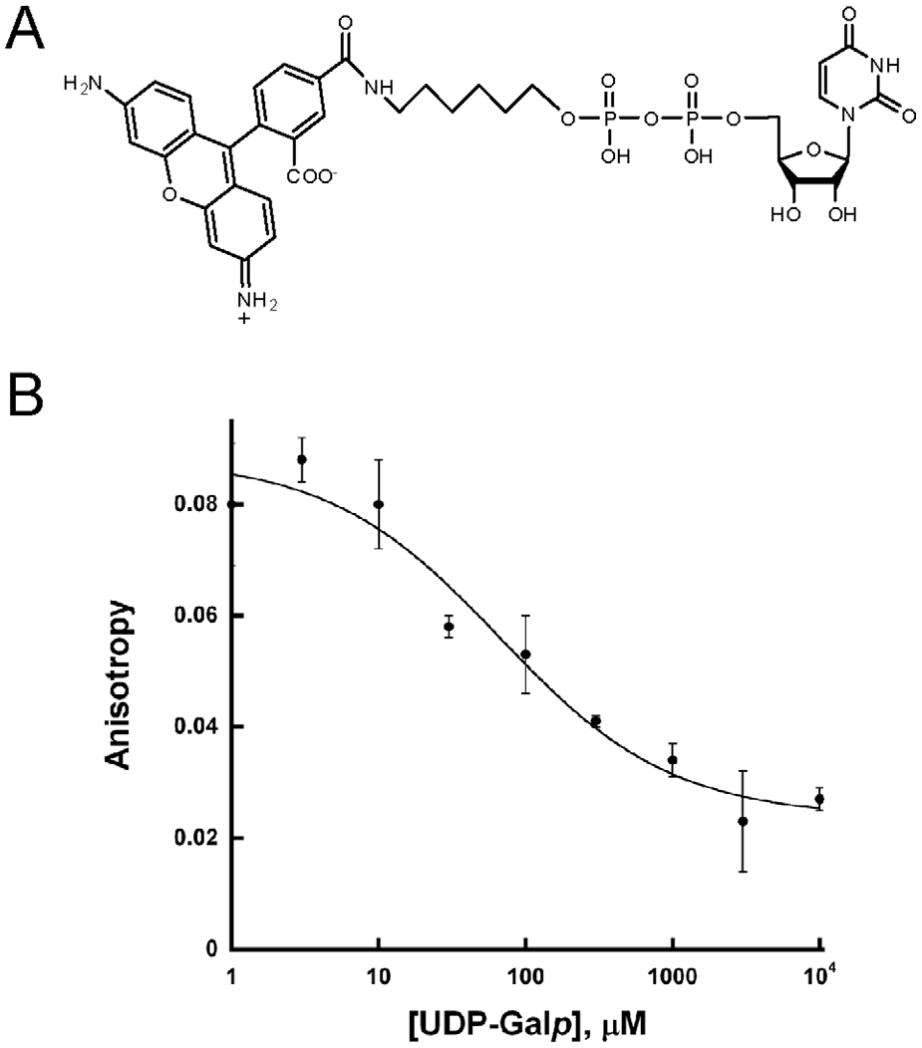
Fluorescence anisotropy assay to measure the affinity of UDP-Gal*p* to TcUGM. A) UDP-rhodamine chromophore used in the fluorescence anisotropy experiments. B) Fluorescence polarization binding assay. The binding of UDP-Gal*p* to chemically reduced TcUGM was monitored by measuring the changes in anisotropy as it displaces UDP-rhodamine from the active site. The K*_d_* values were obtained using equation 3.

To determine if UDP-Gal*p* binds to oxidized or reduced TcUGM, we synthesized UDP-rhodamine to measure binding of the substrate. Fluorescence anisotropy changes induced by release of the bound chromophore to TcUGM upon binding of the substrate can be used to calculate the dissociation constant (Figure 7A) [37].UDP-Gal*p* was found to have very weak affinity to oxidized TcUGM. We tested concentrations of substrate as high as 100 mM and were unable to measure binding. However, when TcUGM was reduced with dithionite, a K*_d_* of 70±40 µM was determined demonstrating UDP-Gal*p* only binds to the reduced state of TcUGM (Figure 7B).

### Isolation of flavin-galactose adduct

**Figure 8:**
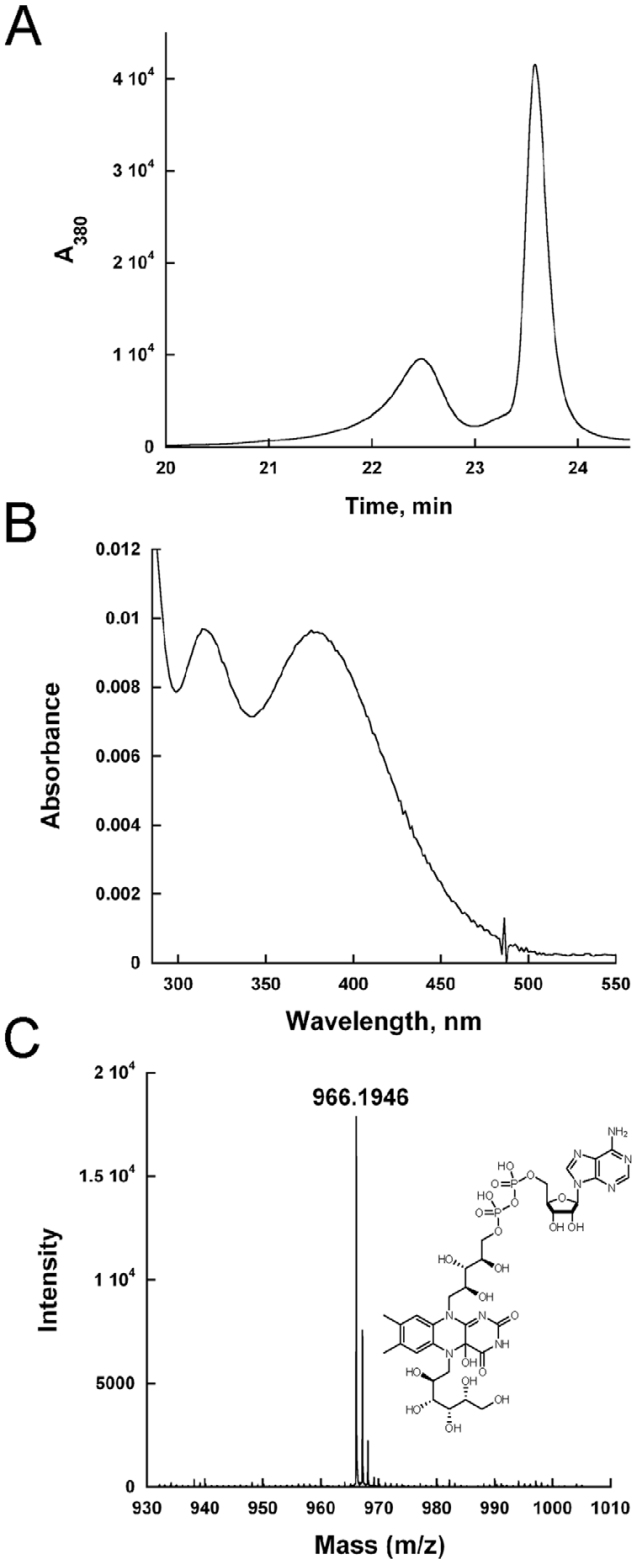
Trapping of a covalent flavin intermediate. A) HPLC traces of the flavin sugar adduct from free FAD. The peak eluding at 22.5 min is the adduct, while the second peak at 23.6 min is FAD. B) Spectrum of the C4a- hydroxyflavin-galactose adduct. C) High resolution mass spectrometry results of the peak containing the flavin adduct. The inset shows the structure of the adduct with a hydroxyl group at the flavin C4a-position.

In the UGM reaction, a novel role for the flavin cofactor has been proposed. It was suggested that the flavin could attack UDP-Gal*p* at the anomeric carbon, leading to cleavage of the C-O bond (Figure 2, **2**). In the bacterial enzymes a flavin-galactose adduct was isolated and characterized, supporting this role for the flavin cofactor [21], [22]. Involvement of a flavin sugar adduct in the reaction of eukaryotic UGMs was investigated. The adduct was trapped by performing activity assays in the presence of sodium cyanoborohydride, which reduces the iminium ion during turnover. The flavin was extracted and then isolated by HPLC (Figure 8A). Two major peaks were observed, one corresponding to native FAD, while the other had spectral peaks at 230 nm, 315 nm, and 378 nm (Figure 8B). This flavin spectrum is consistent with previous published spectra for the N5-flavin adduct however the peak at 378 nm is indicative of a C4a-oxygen-flavin adduct [21], [40]. This compound was characterized by high resolution mass spectrometry (HRMS) and shown to have a mass of 966.1946 Da (Figure 8C). This mass closely corresponds to the expected mass of the FAD-galactose adduct of 950.2340 Da; the difference corresponds to the mass of a hydroxyl group. We propose that the flavin was hydroxylated at the C_4a_ position during the trapping and isolation process, which agrees with the observed spectrum.

### Monitoring the reaction of reduced TcUGM with UDP-Gal*p*, UDP-Gal*f*, UDP, and UDP-Glc

**Figure 9:**
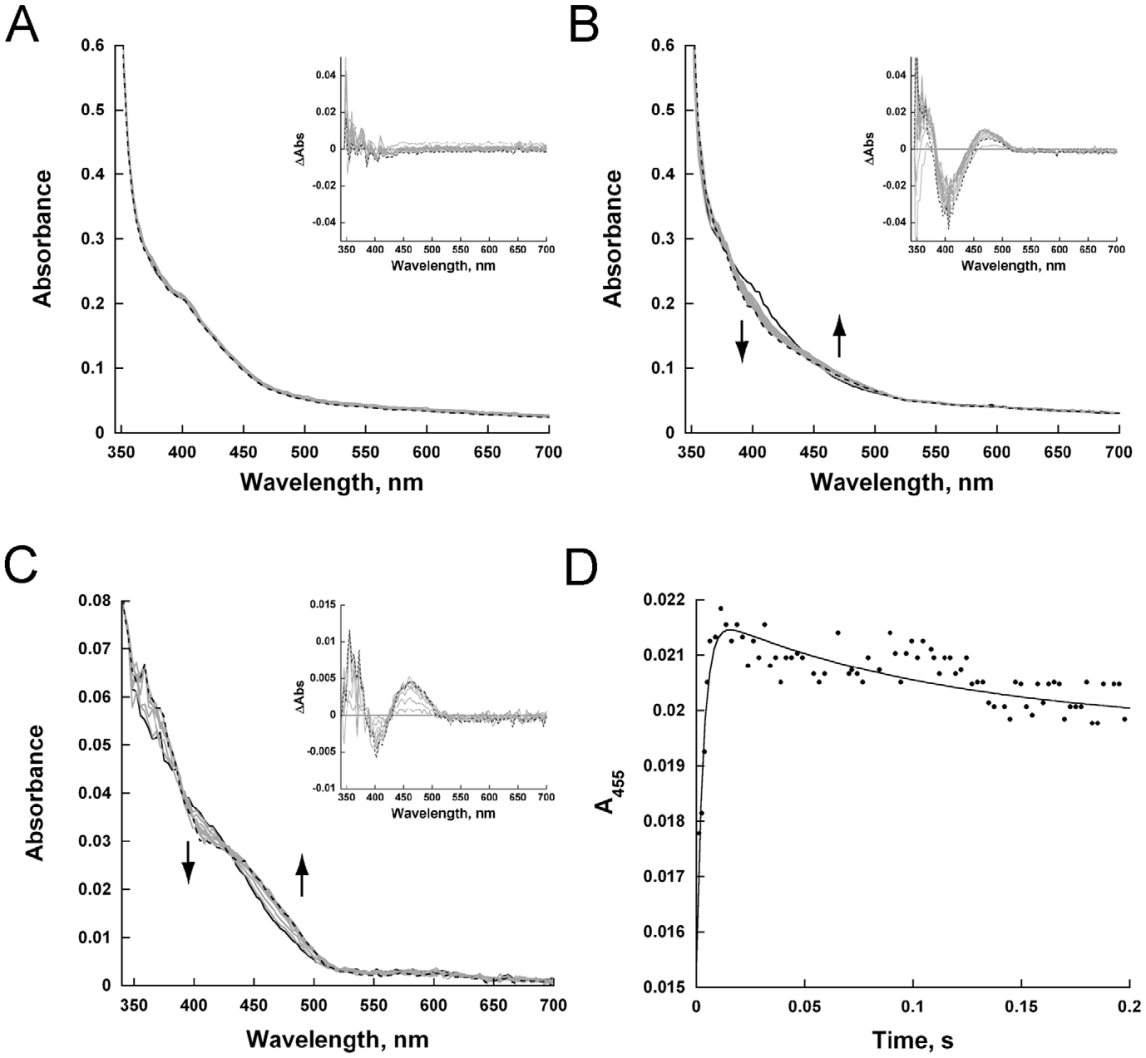
Rapid reaction kinetics with reduced TcUGM mixed with substrate and substrate analogs. The initial spectrum (1.2 ms) is shown as a solid line, the final spectrum (14 ms for UDP-Gal*f* and 1.5 s for all other analogs) as a dashed line, and intermediate time points as gray lines (spectra every 1.26 ms for UDP-Gal*f* and every tenth spectrum collected of 400 points on logarithmic time scale over 2 s for all other analogs). Reduced TcUGM mixed with 0.25 mM UDP (A), 0.25 mM UDP-Gal*p* (B), 0.15 mM UDP-Gal*f* (C), and formation of the iminium ion monitored at 455 nm (D). The data was fit to equation 3.

The results presented in the previous section and the previously published data, establish that a flavin-galactose adduct is formed in the reaction catalyzed by UGMs. However, the mechanism leading to the formation of this intermediate can occur via the formation of a flavin and sugar radical (Figure 2, **6**-**2**) or by a direct nucleophilic attack by the flavin (Figure 2, **1-2**). Since a flavin semiquinone has distinct spectroscopic characteristics, we used rapid reaction kinetics to monitor the reaction at early time points. In this experiment, anaerobically reduced TcUGM was mixed with either buffer alone, UDP, UDP-glucose (UDP-Glc), UDP-Gal*p*, or UDP-Gal*f* in the stopped-flow spectrophotometer and complete spectra were collected between 350 nm and 700 nm (Figure 9). When the enzyme was mixed with buffer alone or with UDP, no major spectral changes were detected (Figure 9A, buffer alone not shown). Difference spectra of TcUGM after mixing with UDP-Glc showed a slight decrease in absorbance at ∼415 nm, which agrees with previous data with UDP-Glc binding to *Klebsiella pneumoniae* UGM (KpUGM) (Figure S3) [21], [41]. TcUGM mixed with either UDP-Gal*p* or UDP-Gal*f* showed the same spectral changes with a decrease at ∼405 nm, an isosbestic point at ∼425 nm (red shifted 10 nm with UDP-Gal*p*), and a peak at ∼460 nm (Figure 9B, C). Equilibrium binding of UDP-Gal*p* to KpUGM showed similar flavin spectral changes, however, it was red shifted by ∼50 nm [21]. The change in absorbance at the higher wavelengths was attributed to the formation of the iminium ion intermediate [21]. As the change at higher wavelengths does not occur upon binding of the known ligand UDP-Glc, but only upon substrate binding, we propose that these spectral changes correspond to the formation of the iminium ion in TcUGM (Figure 2, **3**). A *k_1_* value of 310±40 s^−1^ and a *k_2_* value of 7.9±0.7 s^−1^ were obtained from fitting the absorbance changes at 452 nm to a double exponential equation (Figure 9D). We attributed the fast rate to the formation of the iminium ion. Since the rate of *k_2_* closely matches the value of *k_cat_*, we concluded that it is associated with the rate limiting step, which could be either ring closing or reattachment of UDP. More importantly, upon mixing reduced TcUGM and substrate, a flavin semiquinone was not observed, suggesting either the semiquinone does not form or that the rate of decay is faster than the rate formation.

## Discussion

UGM belongs to a growing group of flavoproteins where the protein environment modulates the reactivity of the flavin to perform novel non-redox chemistries [42], [43]. There are two proposed mechanisms for UGM. The proposed mechanisms differ on the steps leading to the formation of the flavin galactose adduct. In one mechanism, the flavin acts as a nucleophile and attacks the anomeric carbon leading to the formation of the flavin-galactose adduct intermediate, which can either occur via a direct attack on UDP-Gal*p* (S_N_2) or on a postulated oxocarbenium ion intermediate (S_N_1)(Figure 2, **1**-**2**). In the other mechanism, elimination of UDP leads to the formation of an oxocarbenium ion followed by a single electron transfer from the reduced flavin (Figure 2, **6**), forming a flavin seminquinone and sugar radical. Radical rearrangement of the N5 of the flavin and subsequent recombination leads to the formation of the flavin-galactose adduct (Figure 2, **2**). Once the adduct is formed, the galactose ring opens leading to the formation of a sugar-flavin iminium ion (Figure 2, **3**) [21], [22]. After recyclization, the final step in the reaction involves an attack on the anomeric carbon by UDP, leading to the formation of UDP-Gal*f* (Figure 2, **4-5**).

These mechanisms were developed and supported by data obtained entirely from studies on prokaryotic UGMs and several aspects of the mechanism are not completely understood. It was shown by position isotope effects (PIX) that the glycosidic bond between galactose and UDP was broken during catalysis [44]. Later studies demonstrated that the semiquinone of the flavin is stabilized in the presence of UDP and UDP-Gal*p* and that UGM is inactive with 5-dezaflavin, supporting the single electron transfer mechanism [23], [24]. The formation of a flavin N5-C1 galactose adduct was then demonstrated by trapping the immium ion with NaCNBH_3_ [21], [22]. This result supports a mechanism in which the flavin acts as a nucleophile. Furthermore, the structure of bacterial enzymes in complex with UDP-Gal*p*, show proper binding conformation for nucleophilic attack by the flavin cofactor [22], [45]. Also in support of the nucleophilic mechanism, it was also shown that the flavin was present as in the anionic hydroquinone form, a feature that is indicative of the N5 being a good nucleophile [21], [24].

Here, we expressed and purified the eukaryotic UGM from the parasite *T. cruzi*. Recombinant TcUGM was found to function as a monomer in solution, which differs from both the dimeric prokaryotic UGMs, and the tetrameric fungal UGM (AfUGM) [17], [20]. However, it is similar to the parasitic UGM from *L. major* (LmUGM), which is also a monomer [27]. This suggests that TcUGM and LmUGM are closely related, which agrees with their higher percent amino acid identity (60% with LmUGM, compared to 40% with AfUGM and 15% with EcUGM and MtUGM). TcUGM was found to be active only in the reduced form. Chemically reduced TcUGM has similar kinetic parameters with UDP-Gal*f* as substrate as other UGMs [17], [27], [28], [45], [46].

Having stable and active TcUGM, a series of experiments were performed to address several unanswered questions about the chemical mechanism. We sought to determine the source of the reducing equivalents required for catalysis. *In vivo*, a common source of reducing equivalents for flavoenzymes is NAD(P)H. It was determined that recombinant TcUGM could reduced by NADPH or NADH in the absence of UDP-Gal*p*, and was oxidized very slowly by molecular oxygen. In the presence of substrate, the reaction with oxygen was decreased. Thus, substrate binding protects the enzyme from oxidation, which is essential for activity since TcUGM is active only in the reduced form. There is no a canonical NADPH binding domain in the primary sequence of TcUGM, thus the binding site of NADPH remains to be identified. The reactivity of TcUGM with NAD(P)H is unique since prokaryotic UGMs are unable to react with reduced coenzymes. Perhaps the bacterial enzymes have specific redox partners in the cell.

The rate of flavin reduction by NAD(P)H was directly monitored in the stopped-flow spectrophotometer. It was shown that the rate constant of flavin reduction and the affinity for NADPH was higher than for NADH. These results showed a 40-fold higher k*_red_*/K*_d_* value for NADPH over NADH, suggesting that NADPH is the preferred coenzyme.

The rate of flavin reduction was not affected by the presence of UDP-Gal*p*, indicating that binding of UDP-Gal*p* is not a prerequisite for reduction. Furthermore, UDP-Gal*p* only binds to the reduced form of TcUGM, indicating that UDP-Gal*p* binds after flavin reduction. The K*_d_* value of UDP-Gal*p* for the reduced UGM was determined to be 70±40 µM by fluorescence anisotropy. Thus, the kinetic mechanism starts with binding of NADPH followed by hydride transfer and UDP-Gal*p* binding. Binding of UDP-Gal*p* protects the enzyme from reacting with molecular oxygen. It can be estimated from the k*_cat_* value (∼12 s^−1^) and the rate of oxidation, (0.01 s^−1^), that for every NADPH oxidized the enzyme can turn over ∼1000 times.

When reduced TcUGM was mixed with UDP-Gal*p* or UDP-Gal*f* using stopped-flow spectroscopy only a small decrease in absorbance at 415 nm and an increase at 460 nm was observed. These changes were not observed when the enzyme was mixed with either UDP or UDP-Glc, ligands that do not turn over. Therefore, we assigned the spectral changes to the formation of the iminium ion. Similar spectral changes have been reported with bacteria UGM under equilibrium conditions, which are consistent with our conclusion [21]. Interestingly, the flavin semiquinone was not observed suggesting either it does not occur or it decays rapidly preventing detection. By addition of sodium cyanoborohydride into the reaction mixture, a flavin-sugar adduct was trapped and was isolated and characterized by mass spectroscopy. This result validates the assignment of the iminium ion intermediate observed in the stopped-flow experiments. This adduct has also been characterized in KpUGM as a flavin N5-C1 galactose intermediate by HRMS and ^1^H NMR [21], [22]. From our rapid reaction kinetics data, the changes in flavin absorbance best fit a double exponential equation with a fast and slow rate. We assign the fast rate to the formation of the iminium ion, which occurs at 310±42 s^−1^. While the slow rate occurred at 7.9±0.7 s^−1^, and as it closely matches the k*_cat_* value (∼12 s^−1^), we propose that this corresponds to the rate-limiting step. One possibility is that the rate constant that controls k*_cat_* corresponds to product release. To determine if product release is the rate-limiting step in the reaction, viscosity effects studies on k*_cat_* were performed. If product release is the slow step that determines the k*_cat_* value, a linear decrease in the k*_cat_* value should be observed as a function of increasing concentrations of viscogen. Using glycerol as a viscogen, no changes in the k*_cat_* value was observed, clearly indicating that product release is not the rate-limiting step in the reaction of TcUGM. Therefore, we conclude that the rate-limiting step must be ring closing or UDP reattachment. Further studies are needed to elucidate between the two.

Our results from the rapid reaction kinetic analysis show that a flavin iminium ion is formed and suggest that a flavin semiquinone does not play a role in catalysis. Furthermore, we were able to trap and characterize a flavin-galactose adduct. These data strongly supports the role of the flavin as a nucleophile. We proposed that the formation of this adduct occurs via a direct attack of the flavin (S_N_2 mechanism) rather than formation of an oxocarbenium ion, followed by flavin attack (S_N_1 mechanism). Our conclusion is supported by data from Sun et al. where linear free energy relationship (LFER) studies with prokaryotic UGM reconstituted with various FAD analogs show changes in k*_cat_* values that correlate linearly with changes in the nucleophilicity of the flavin N5 (ρ value of −2.4), which is consistent with an S_N_2 mechanism [47]. Also, previous studies with *E. coli* UGM, it was shown that UDP-[2-deoxy-2-fluoro]Gal*f* functions as a substrate with a k*_cat_* value 10^3^ slower than the reported *k_cat_* with UDP-Gal*f* [28]. In contrast, in studies with glycosyltransferases, where the formation of an oxocarbenium ion is known to occur, it has been shown that substrates with fluorine at the C2 position destabilize the formation of the oxocarbenium ion preventing the reaction from occurring [48]–[51]. Therefore, if the UGM reaction proceeded by an oxocarbenium ion, UDP-[2-deoxy-2-fluoro]Gal*f* should act as an inhibitor instead of as a slow substrate.

**Figure 10:**
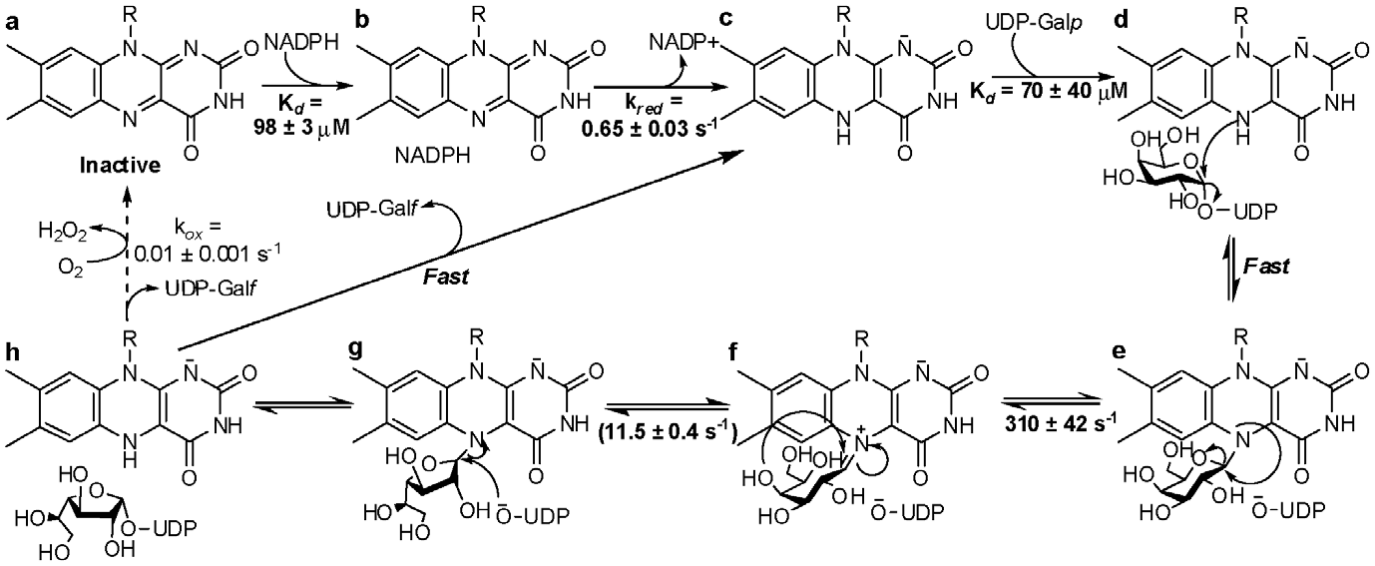
Chemical mechanism of TcUGM. The reaction requires the oxidized flavin cofactor (**a**) to be reduced for activity. First, NADPH binds to the oxidized enzyme (**b**), and only after the flavin is reduced (**c**) will UDP-Gal*p* bind (**d**). The flavin then acts as a nucleophile attacking the C1 of galactose and forming a flavin sugar adduct (**e**), which occurs rapidly (**f**). This is followed by ring opening and recyclization (**g**). The rate limiting step in the reaction corresponds to either galactose isomerization or reattachment of the UDP (**f** to **g**). We postulate that the rate limiting step is the isomerization step. The final step is release of UDP-Gal*f*, which occurs rapidly. The enzyme can proceed to the next reaction cycle or be slowly oxidized by molecular oxygen (**h** to **a**).

Together, the data presented here provides a detailed description of the mechanism of the parasitic TcUGM, which is summarized in Figure 10. We show that TcUGM first binds to NADPH (Figure 10, **b**), and after hydride transfer (Figure 10, **c**), UDP-Gal*p* binds (Figure 10,**d**). We propose that an adduct forms by direct attack of the flavin to the C1 position of galactose (Figure 10, **e**), without the formation of a flavin semiquinone or oxocarbenium ion. Ring opening leads to the rapid formation of an iminium ion (Figure 10, **f**). Product release was determined not to be rate limiting, therefore, ring closing or formation of the glycosidic bond between Gal*f* and UDP represent the rate determining step(s) in this reaction. Since UDP is predicted to remain bound in the active site, we believed that formation of the anomeric bond will be fast, therefore, the slow step is most probably the isomerization step (Figure 10, **f** to **g**). The mechanism of TcUGM clearly shows a unique mode of action for the flavin cofactor, where it functions as a nucleophile and a scaffold for holding the reaction intermediates. These results will aid in the development of new drugs against human pathogens such as *T. cruzi*, *A. fumigatus*, and *L. major*.

## Supporting Information

File S1 **Synthesis of UDP-Gal** ***f*** **.**
(DOC)

Figure S1 **Flavin spectrum and SDS-PAGE of purified TcUGM.**
(TIF)

Figure S2
**Size exclusion chromatography of TcUGM.** The standards aprotinin (1, 6.5 kDa), ribonuclease (2, 13 kDa), ovalbumin (3, 43 kDa), conoalbumin (4, 75 kDa), aldolase (5, 158 kDa), and ferritin (6, 440 kDa) were used to calculate the K_av_ values using the equation, 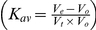, where V_o_ is the void volume of the column; V_t_ is the total volume of the column and V_e_ is the elution volume of the protein). TcUGM is shown on the plot as a black square.(TIF)

Figure S3 Rapid reaction kinetics with reduced TcUGM mixed with 0.25 mM UDP-Glc. Inset show the difference spectra.
(TIF)
