# Supplementary material for: Chemical Mechanism of UDP-Galactopyranose Mutase from Trypanosoma cruzi: A Potential Drug Target against Chagas' Disease
Source: PLoS One. 2012 Mar 20;7(3):e32918. doi: 10.1371/journal.pone.0032918 (PMC3308961; doi:10.1371/journal.pone.0032918)
Supplement: File S1 — Synthesis of UDP-Gal f . (DOC) [file pone.0032918.s001.doc]

**Synthesis of UDP-Gal*f*.**

*Materials.* All reagents and solvents used in the synthesis, if not specified otherwise, were obtained from Sigma-Aldrich (St. Louis, MO) or Fisher Scientific (Pittsburgh, PA) and were used without further purification. All reactions were monitored to completion using thin layer chromatography (TLC) with p-anisaldehyde staining for visualizing carbohydrates along with UV detection when possible. ^1^H and ^13^C NMR were recorded on a 400MR Varian-400 Hz spectrometer. CHCl_3_ (7.27 ppm) or HOD (4.79 ppm) were used as an internal reference.

*Step 1: Synthesis of 1,2,3,5,6-Penta-O-Acetyl-α/β-D-Galactofuranoside (2).* The method was adapted with slight modifications from previously published protocols.^1-3^ D-galactose (**1**) (10.0 g, 55 mmol) was dissolved in anhydrous pyridine (200 mL) and stirred at 110 °C for 2 h. Then, acetic anhydride was added in one portion (60 mL) through the condenser, the temperature was slowly lowered to room temperature, and the reaction continued stirring overnight. The dark reaction mixture was poured on ice (700 g) while stirring and when the ice melted, the oily residue was extracted with methylene chloride (3 × 300 mL), dried with anhydrous sodium sulfate, and concentrated to yield a crude mixture of galactopyranosides and galactofuranosides as a yellow oil. Most of the galactopyranose products (8.9 g, 23%) were precipitated out of the crude mixture as a white solid by crystallizing the crude oil from ethanol. The filtrate was concentrated to yield oily residue that consisted of a mixture of protected *α*- and *β*-galactofuranosides **2** (*α*/*β*:1/1, 5.2 g, 13%). A repeated crystallization was needed if galactopyranose products were still detected in the filtrate. NMR data were in agreement with previously published data.^4,5^ ^1^H-NMR (CDCl_3_, 400 MHz, *α* and *β* isomers): δ = 2.04-2.16 (m, 30H, *CH_3_*CO), 4.13-4.24 (m, 2H), 4.29-4.38 (m, 4H), 5.10 (d, J = 5.4 Hz, 1H), 5.18 (bs, 1H), 5.25-5.38 (m, 2H), 5.34-5.39 (m, 1H), 5.56 (t, J = 6.5 Hz, 1H), 6.19 (s, 1H, 1-H*^β^*), 6.33 (d, J = 4.1 Hz, 1H, 1-H*^α^*). ^13^C-NMR (CDCl_3_, 400 MHz, *α* and *β* isomers): δ = 20.47, 20.64, 20.67, 20.70, 20.79, 20.82, 21.00, 21.04, 62.11, 62.57, 69.25, 70.34, 73.41, 75.31, 76.33, 79.09, 80.60, 82.14, 93.09*^α^*, 99.12*^β^*, 169.04, 169.41, 169.77, 170.01, 170.53.

*Step 2: Synthesis of Dibenzyl 2,3,5,6-tetra-O-Acetyl-α-D-Galactofuranosyl Phosphate (3).* The method was adapted with slight modifications from previously published protocols.^3,6^ The solution of peracetylated *α*-Gal*f* **2** (α/β mixture, 1.0 g, 2.6 mmol) in anhydrous dichloromethane (10 mL) was cooled to 0 °C in an ice bath followed by the addition of 33% HBr in glacial acetic acid (2.0 mL). The resulting reddish solution was stirred for 2 h maintaining 0 °C temperature until TLC analysis (ethyl acetate/hexane – 1/1, stained with p-anisaldehyde) showed the absence of the starting material. The solvent and residual acids were removed by repeatedly co-evaporating the mixture with anhydrous toluene. The residue was re-dissolved in 20 mL of toluene, followed by the addition of triethylamine (0.8 mL, d = 0.72 g/mL, 7.8 mmol) and dibenzyl phosphate (1.0 g, 3.6 mmol), and the resulting mixture was stirred for 1 h at 0 °C and overnight at room temperature. The white precipitate of triethylammonium bromide was filtered off and the filtrate concentrated and purified by flash column chromatography (eluent: ethyl acetate/hexane – 1/1). The product, *α*-Gal*f* 1-phosphate **3** was found in the last fractions and upon concentration yielded a yellowish oil (0.6 g, 40%). ^1^H-NMR (CDCl_3_, 400 MHz): δ = 1.93 (s, 3H, CO*CH_3_*), 1.96 (s, 3H, CO*CH_3_*), 2.01 (s, 3H, CO*CH_3_*), 2.06 (s, 3H, CO*CH_3_*), 4.10 (dd, J = 6.3, 11.9 Hz, 1H), 4.21 (dd, J = 4.4, 7.1 Hz, 1H), 4.28 (dd, J = 4.7, 11.9 Hz, 1H), 5.05 (d, J = 7.6, 4H, *CH_2_*Bn), 5.14-5.26 (m, 2H), 5.44-5.58 (m, 1H), 5.97 (dd, J = 4.4, 5.6 Hz, 1H), 7.28-7.36 (m, 10H, arom.).

*Step 3. Synthesis of α-D-Galactofuranosyl bis(triethylammonium) phosphate (4).* The method was adapted with slight modifications from previously published protocols.^3,6,7^ To the solution of peracetylated *α*-Gal*f* 1-phosphate **3** (240 mg, 0.395 mmol) in ethyl acetate/ethanol (1/1, 4 mL) triethylamine (0.4 mL) and Pd/C (10_wt_%) (40 mg) were added, and the reaction was hydrogenated at room temperature and atmospheric pressure for 24 h. The mixture was filtered through the celite plug and concentrated to yield ayellowish oil. Debenzylated product, without further purification, was dissolved in methanol/water/triethylamine (2 mL/0.8 mL/0.4 mL) and the resulting solution was stirred at room temperature overnight, then concentrated to yield slightly yellowish oil, which was pure *α*-Gal*f* 1-phosphate **4** as a bis(triethylammonium) salt (158 mg, 86%). ^1^H-NMR (D_2_O, 400 MHz): δ = 1.29 (t, J = 7.3 Hz, 18H, *CH_3_*CH_2_N), 3.09 (q, J = 7.3 Hz, 12H, CH_3_*CH_2_*N), 3.59-3.83 (m, 4H), 4.04-4.15 (m, 1H), 4.17-4.25 (m, 1H), 5.50 (t, J = 4.7 Hz, 1H). NMR data were in agreement with those published.^6,7^

*Step 4: Chemienzymatic coupling of UDP to α-Galf-1-Phosphate.* UDP-Gal*f* was synthesized using the previously published chemienzymatic method as reported by the Lowary lab without modification.^8^ ^1^H-NMR (D_2_O, 400 MHz): δ = 3.63-3.70 (m, 1H), 3.72-3.77 (m, 1H), 3.78-3.83 (m, 1H), 3.84-3.88 (m, 1H), 4.16-4.33 (m, 4H), 4.38-4.44 (m, 2H), 5.65-5.69 (m, 1H), 5.96-6.06 (m, 2H), 7.98 (d, J = 8.1 Hz, 1H).

**References**

(1) Cattiaux, L.; Sendid, B.; Collot, M.; Machez, E.; Poulain, D.; Mallet, J. M. *Bioorg Med Chem* **2011**, *19*, 547-55.

(2) D'Accorso, N.; Thiel, I. M. E.; Schüller, M. *Carbohydr Res* **1983**, *124*, 177-184.

(3) Zhang, Q.; Liu, H. *J Am Chem Soc* **2001**, *123*, 6756-66.

(4) Davis, C. B., Griffith University, 2006.

(5) Ness, R. K.; Fletcher, H. G.; Hudson, C. S. *J Am Chem Soc* **1951**, *73*, 3742-3744.

(6) Tsvetkov, Y. E.; Nikolaev, A. V. *J Chem Soc, Perkin Transactions 1* **2000**, 889-891.

(7) de Lederkremer, R. M.; Nahmad, V. B.; Varela, O. *J Org Chem* **1994**, *59*, 690-692.

(8) Poulin, M. B.; Lowary, T. L. *Methods Enzymol*, *478*, 389-411.
