# Supplementary figures and images for: Chemical Mechanism of UDP-Galactopyranose Mutase from Trypanosoma cruzi: A Potential Drug Target against Chagas' Disease
Source: PLoS One. 2012 Mar 20;7(3):e32918. doi: 10.1371/journal.pone.0032918 (PMC3308961; doi:10.1371/journal.pone.0032918)

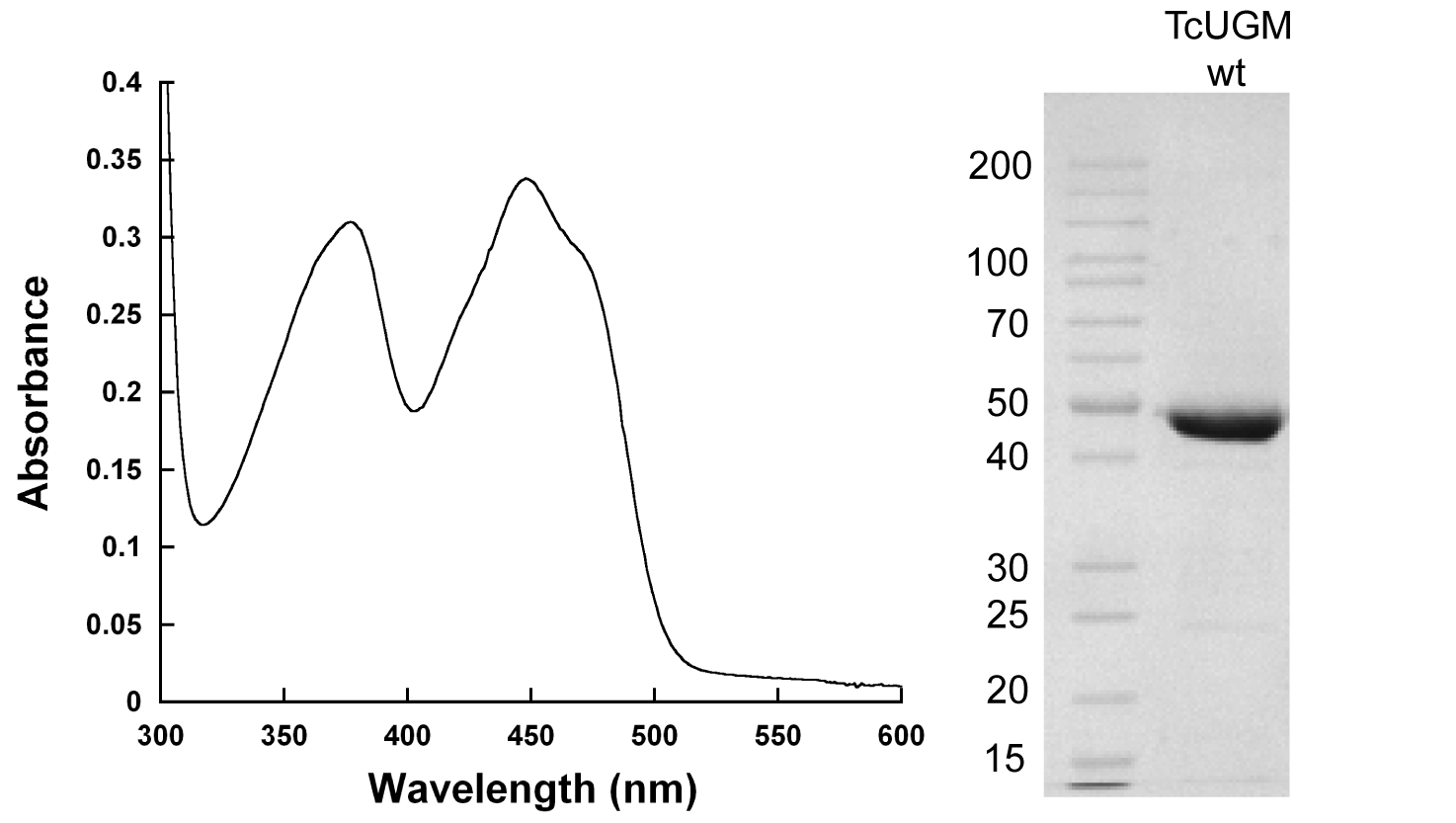

Supplement: Figure S1 — Flavin spectrum and SDS-PAGE of purified TcUGM. (TIF) [file pone.0032918.s002.tif]

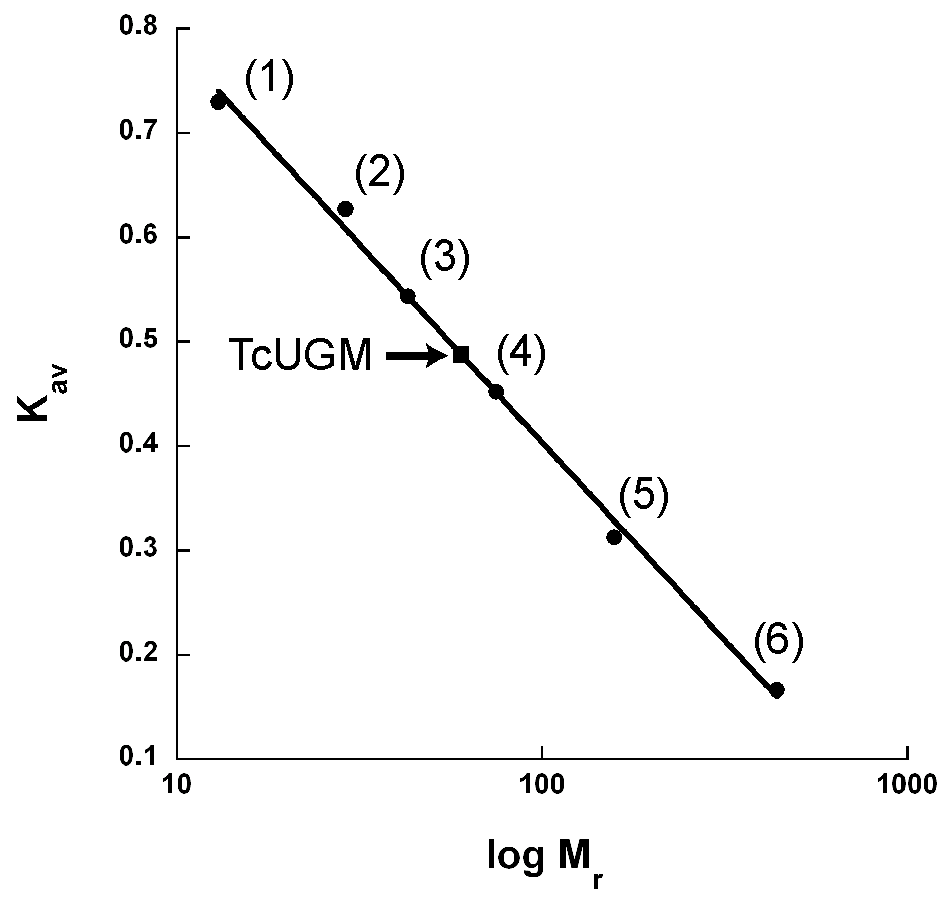

Supplement: Figure S2 — Size exclusion chromatography of TcUGM. The standards aprotinin (1, 6.5 kDa), ribonuclease (2, 13 kDa), ovalbumin (3, 43 kDa), conoalbumin (4, 75 kDa), aldolase (5, 158 kDa), and ferritin (6, 440 kDa) were used to calculate the Kav values using the equation, , where Vo is the void volume of the column; Vt is the total volume of the column and Ve is the elution volume of the protein). TcUGM is shown on the plot as a black square. (TIF) [file pone.0032918.s003.tif]

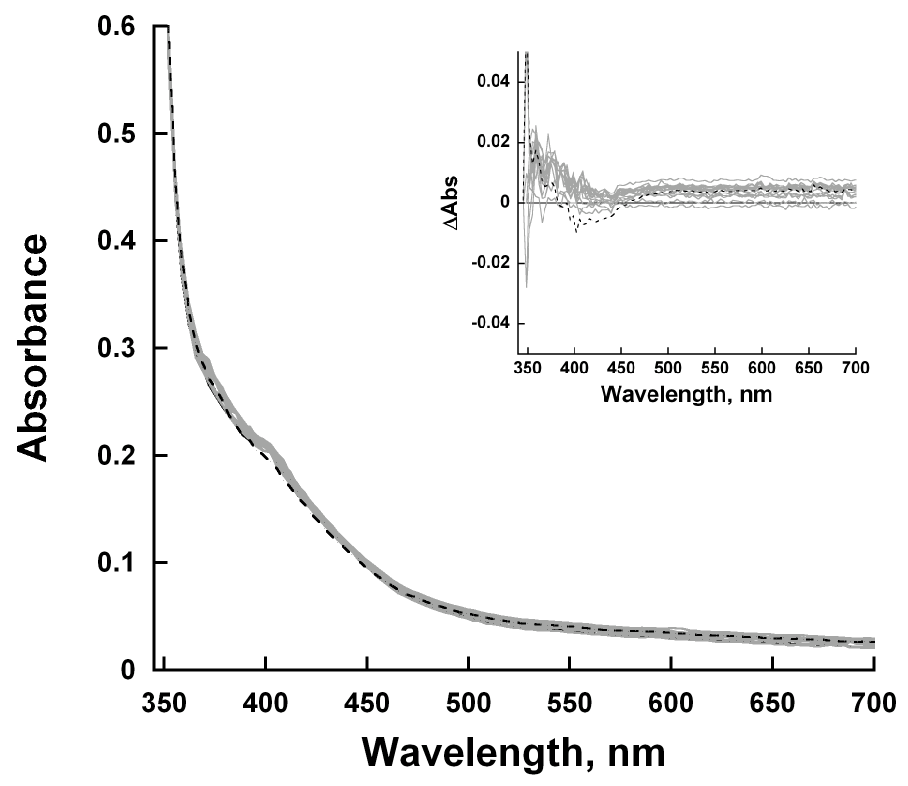

Supplement: Figure S3 — Rapid reaction kinetics with reduced TcUGM mixed with 0.25 mM UDP-Glc. Inset show the difference spectra. (TIF) [file pone.0032918.s004.tif]
